# Dietary methionine source alters the lipidome in the small intestinal epithelium of pigs

**DOI:** 10.1038/s41598-022-08933-3

**Published:** 2022-03-22

**Authors:** Isabel I. Schermuly, Stella Romanet, Martina Klünemann, Lucia Mastrototaro, Robert Pieper, Jürgen Zentek, Rose A. Whelan, Jörg R. Aschenbach

**Affiliations:** 1 Institute of Veterinary Physiology, Freie Universität Berlin, Oertzenweg 19b, 14163 Berlin, Germany; 2 Evonik Operations GmbH, Animal Nutrition Services, Hanau-Wolfgang, Germany; 3 Institute of Animal Nutrition, Freie Universität Berlin, Berlin, Germany

**Keywords:** Physiology, Gastroenterology

## Abstract

Methionine (Met) as an essential amino acid has key importance in a variety of metabolic pathways. This study investigated the influence of three dietary Met supplements (0.21% L-Met, 0.21% DL-Met and 0.31% DL-2-hydroxy-4-(methylthio)butanoic acid (DL-HMTBA)) on the metabolome and inflammatory status in the small intestine of pigs. Epithelia from duodenum, proximal jejunum, middle jejunum and ileum were subjected to metabolomics analysis and qRT-PCR of caspase 1, NLR family pyrin domain containing 3 (NLRP3), interleukins IL1β, IL8, IL18, and transforming growth factor TGFβ. Principal component analysis of the intraepithelial metabolome revealed strong clustering of samples by intestinal segment but not by dietary treatment. However, pathway enrichment analysis revealed that after L-Met supplementation polyunsaturated fatty acids (PUFA) and tocopherol metabolites were lower across small intestinal segments, whereas monohydroxy fatty acids were increased in distal small intestine. Pigs supplemented with DL-HMTBA showed a pronounced shift of secondary bile acids (BA) and sphingosine metabolites from middle jejunum to ileum. In the amino acid super pathway, only histidine metabolism tended to be altered in DL-Met-supplemented pigs. Diet did not affect the expression of inflammation-related genes. These findings suggest that dietary supplementation of young pigs with different Met sources selectively alters lipid metabolism without consequences for inflammatory status.

## Introduction

Methionine (Met) is of great importance in the nutrition of livestock. In pigs, it is the second performance-limiting amino acid^1^ and is essential for efficient growth performance. Besides the economic relevance, balanced amino acid supplementation is a key element for reducing the crude protein level in feed and thus agricultural nitrogen emissions. Vice versa, low crude protein diets need to be supplemented with essential limiting amino acids, especially Met, to meet the amino acid requirements of pigs^2^. In Europe, the most common authorized feed additives are DL-Met, L-Met and the hydroxyl analog DL-2-hydroxy-4-methylthiobutyrate (DL-HMTBA) (Reg (EC) No 1831/2003)^1^. Some studies state an equal bioavailability of these Met sources^3,4^, whereas more recent studies show relatively lower bioavailability of DL-HMTBA compared to L- or DL-Met sources^5,6^. When supplemented on equimolar levels, several studies did not identify a difference between supplements in performance parameters^5,7^ or, as shown in cherry valley ducks, carcass traits^8^.

Across vertebrates, Met is a nutritionally essential amino acid with key functions in several biological processes^9^. In the intestinal epithelium, Met is involved in transsulfuration, transmethylation and transamination reactions^10^. The Met derivate S-adenosylmethionine represents a methyl group donor^11^, which is particularly important for DNA methylation^12^, as well as metabolism of neurotransmitters^13,14^ and phosphatidylcholines^15^. Additionally, Met plays a significant role in antioxidant defense through its own antioxidative capacity and as a precursor of cysteine and glutathione^16,17^. The amino acid taurine derived from cysteine is essential for bile acid (BA) conjugation^14^.

A significant portion of dietary Met (~ 20–30%) is metabolized in the intestinal epithelium^18–20^. L-Met is the biologically active form, while D-Met and DL-HMTBA have to be converted to L-Met first^21^. The common intermediate metabolite for L-Met synthesis is 2-keto-4 (methylthio) butanoic acid (KMB), which is synthesized from D-Met by D-amino acid oxidase, from D-HMTBA by D-2-hydroxyacid dehydrogenase and from L-HMTBA by L-2-hydroxy acid oxidase^21^. Subsequent transamination reactions convert KMB into L-Met^21^. Central organs for D-Met and DL-HMTBA metabolization are liver and kidney; however, some metabolization also occurs in other organs like stomach (DL-HMTBA) and small intestine (D-Met)^22^.

Using the same pigs as in the present study, we had identified earlier that pre-feeding with DL-Met increased L-Met absorption in the porcine small intestine, especially in the middle jejunum^23^. Giving the involvement of the small intestine in Met metabolism and additional effects of Met source on absorptive efficiencies, it seemed fair to hypothesize that different dietary Met sources may alter the intracellular metabolism of Met with possible outreach on other metabolic pathways. Therefore, one aim of the current study was to unravel the consequences of different Met supplements on the small intestinal metabolome of pigs.

Given the mutual interactions between feed, gastrointestinal mucosa and microbiota^24,25^, it was further of interest if the aforementioned Met sources may have different effects on gut health. In growing pigs, gut health is crucial for fattening performance and subsequently for economic efficiency as chronic subclinical inflammation inhibits body weight gain^26,27^ and impacts small intestinal function^28^. Several studies investigated the effect of different Met sources on gut health, linking it to possible oxidative stress and inflammation^7,29^. A positive effect of Met supplementation was mostly attributed to improved anti-oxidative status^30,31^. However, Met can also have adverse effects if supplemented in excessive amounts. Based on animal experiments, Met was even described as the most toxic amino acid if overdosed^32^. Therefore, the second aim of the study was to assess the effects of the three different Met supplements on the intestinal inflammatory status as one key readout for gut health.

To achieve the two aims, we used the same pigs as in our previous study and performed a detailed metabolomics analysis on epithelia in four small intestinal segments, i.e. duodenum (DUO), proximal jejunum (PJ), middle jejunum (MJ) and ileum (ILE). The pigs had been supplemented with either 0.21% L-Met, 0.21% DL-Met or 0.31% DL-HMTBA to achieve equivalent Met supplementation levels based on their respective equimolar bioavailability^23^. This resulted in a two-factorial design with the factors intestinal region (DUO, PJ, MJ and ILE) and dietary treatment (L-Met, DL-Met and DL-HMTBA). To include the assessment of Met supplements on porcine intestinal health, we accompanied the metabolomics analyses by mRNA expression analyses of inflammation-related genes.

## Results

### Metabolomics

**Figure 1:**
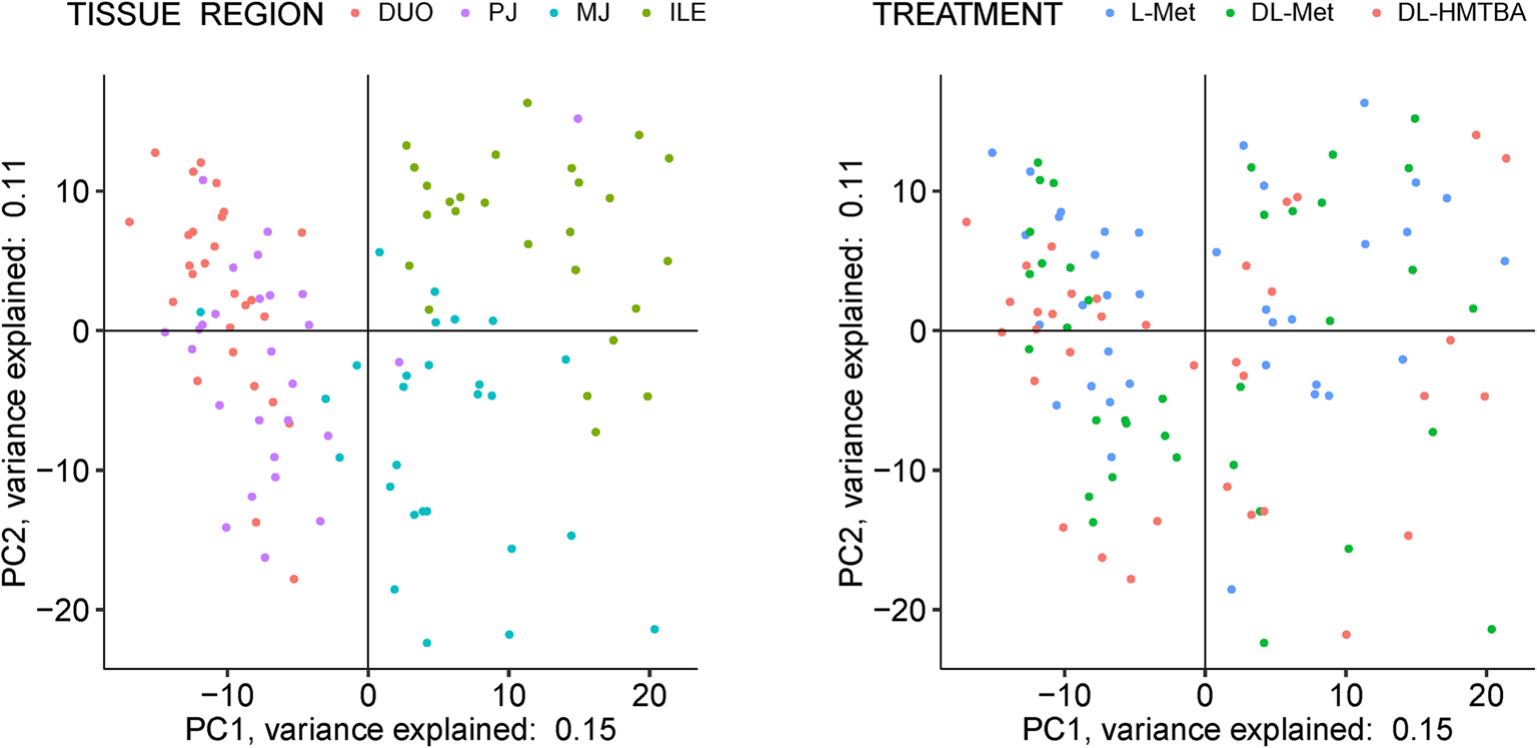
Principal component analysis of samples analyzed by (**A**) tissue region (including all dietary treatments) and (**B**) treatment (including all intestinal regions). Each dot represents one sample. Samples with similar characteristics group together. Different colors represent the four intestinal regions in panel (**A**) (*DUO* duodenum, *PJ* proximal jejunum, *MJ* middle jejunum, *ILE* ileum) and the three feeding groups in panel (**B**) (L-Met; DL-Met; DL-HMTBA).

The metabolomics dataset covered 749 compounds of known identity (Box Plots in Supplementary Table 1). To get an overview on data structure, principal component analysis was performed with the factors intestinal tissue region and dietary treatment. Metabolites clearly separated by the investigated tissue regions. However, clustering by dietary treatment could not be observed (Fig. 1).

Upon two-way ANOVA, 632 metabolites showed main effects of region and 26 metabolites tended to show main effects of region (Pathway Heat Map in Supplementary Table 1). Main effects of treatment were detected for 39 metabolites and another 48 metabolites tended to show treatment effects (Pathway Heat Map in Supplementary Table 1). A region × treatment interaction was observed for 14 metabolites and, as a trend, for another 20 metabolites (Pathway Heat Map in Supplementary Table 1).

Metabolites showing main effects of treatment, region × treatment interactions or trends thereof were included in a pathway enrichment analysis to elucidate changes in metabolic pathways that could be related to the dietary treatment (Table 1). Significantly enriched pathways were polyunsaturated fatty acids (PUFA, n3 and n6; *P* < 0.001), monohydroxy fatty acid metabolism (*P* = 0.026) and secondary bile acid metabolism (*P* = 0.035). Pathways that tended to be altered by treatment were sphingosine metabolism (*P* = 0.064), tocopherol metabolism (*P* = 0.064) and histidine metabolism (*P* = 0.067).

**Table 1:** Results of pathway enrichment analysis. Depicted are the top 9 sub-pathways with *P* ≤ 0.16 and at least two significant metabolites in pathway.

Pathway	Metabolites in pathway	Significant metabolites in pathway	*P*-value
Polyunsaturated fatty acid (n3 and n6)	15	13	< 0.001
Fatty acid, monohydroxy	12	5	0.026
Secondary bile acid metabolism	17	6	0.035
Sphingosines	3	2	0.064
Tocopherol metabolism	3	2	0.064
Histidine metabolism	15	5	0.067
Methionine, cysteine, SAM and taurine metabolism	23	6	0.13
Monoacylglycerol	19	5	0.16
Sterol	5	2	0.16

*SAM* S-adenosylmethionine.

**Figure 2:**
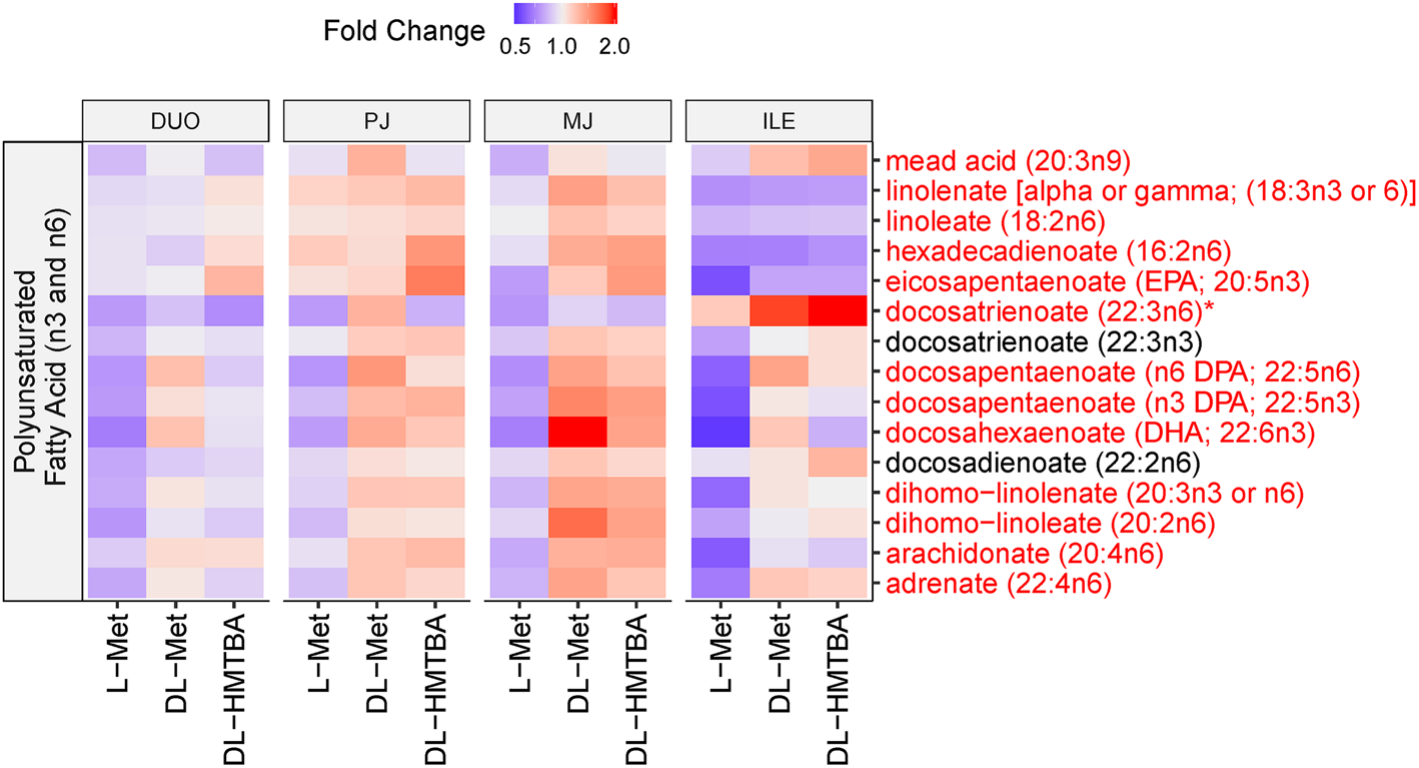
Heat map of the polyunsaturated fatty acids pathway, showing the distribution of all measured pathway-related metabolites along duodenum (DUO), proximal jejunum (PJ), middle jejunum (MJ) and ileum (ILE) in pigs pre-fed with L-Met, DL-Met or DL-HMBTA. The heat map represents the fold changes across tissues as metabolites, which were scaled across all samples. Blue color indicates lower abundance whereas red color indicates higher abundance of metabolites. Red writing of individual metabolites indicates a treatment effect (*P* < 0.10).

The PUFA pathway was most prominently influenced by the dietary treatment (*P* < 0.001; Fig. 2) because all but 2 included metabolites showed main effects of treatment (*P* < 0.05) or trends thereof (*P* < 0.1) with no region × treatment interaction. The PUFA content was generally higher in DL-Met and DL-HMTBA-fed animals compared to L-Met feeding across intestinal regions but particularly obvious in the two jejunal segments (PJ and MJ) and partly in ILE.

**Figure 3:**
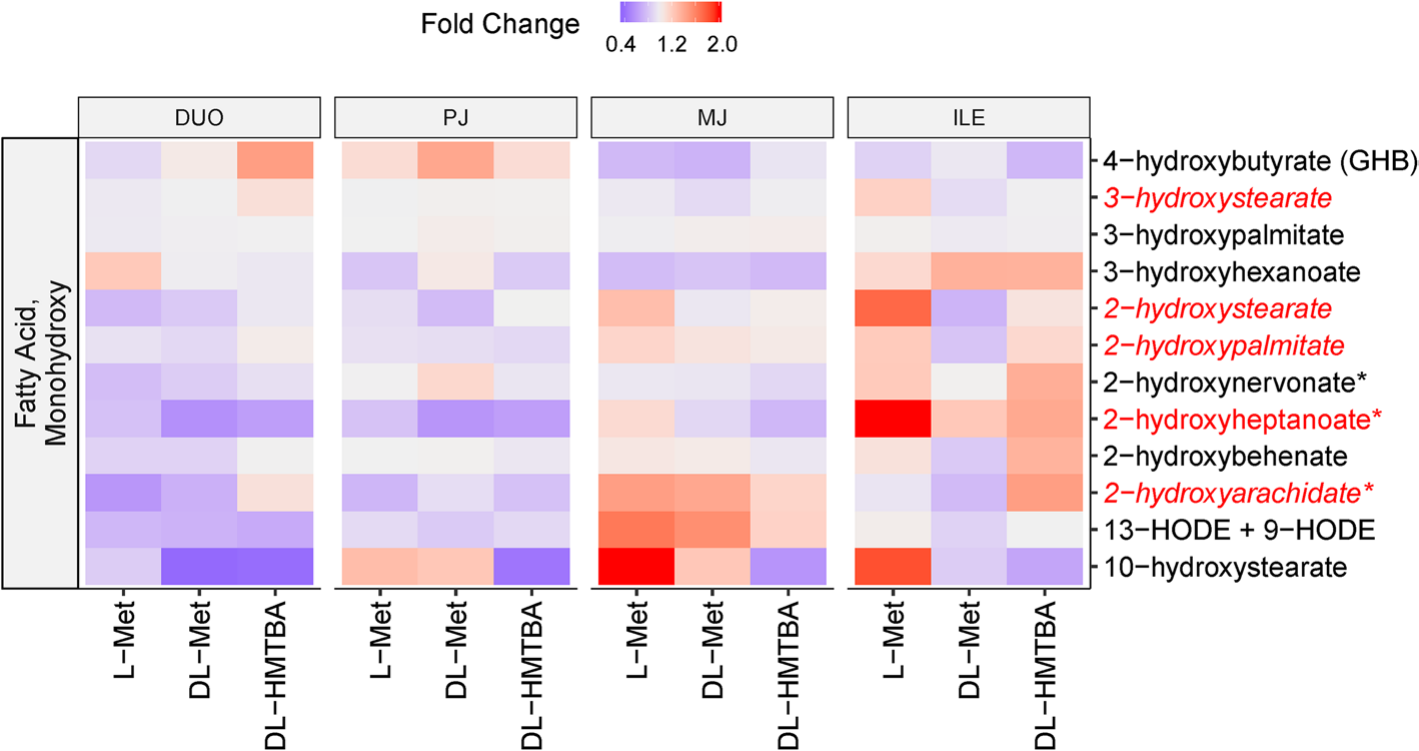
Heat map of the monohydroxy fatty acids pathway, showing the distribution of all measured pathway-related metabolites along duodenum (DUO), proximal jejunum (PJ), middle jejunum (MJ) and ileum (ILE) in pigs pre-fed with L-Met, DL-Met or DL-HMBTA. Blue color indicates lower abundance whereas red color indicates higher abundance of metabolites. Red writing of individual metabolites indicates a treatment effect or, if combined with italics, a region × treatment interaction (*P* < 0.10).

In the significantly enriched monohydroxy fatty acids pathway (*P* = 0.026), 1 out of 12 monohydroxy fatty acids showed a treatment main effect (*P* < 0.05) and 4 showed a region × treatment interaction or trends thereof (*P* < 0.1; Fig. 3), the latter indicating that treatment affected monohydroxy fatty acid abundance selectively in certain intestinal regions. A steep increase of several monohydroxy fatty acids was observed for pigs fed L-Met in ILE and partly MJ. Pigs from the DL-HMTBA feeding group showed an increase of monohydroxy fatty acids only in ILE. Accordingly, DL-Met-fed animals had the lowest abundance of several monohydroxy fatty acids in the more distal small intestinal segments.

**Figure 4:**
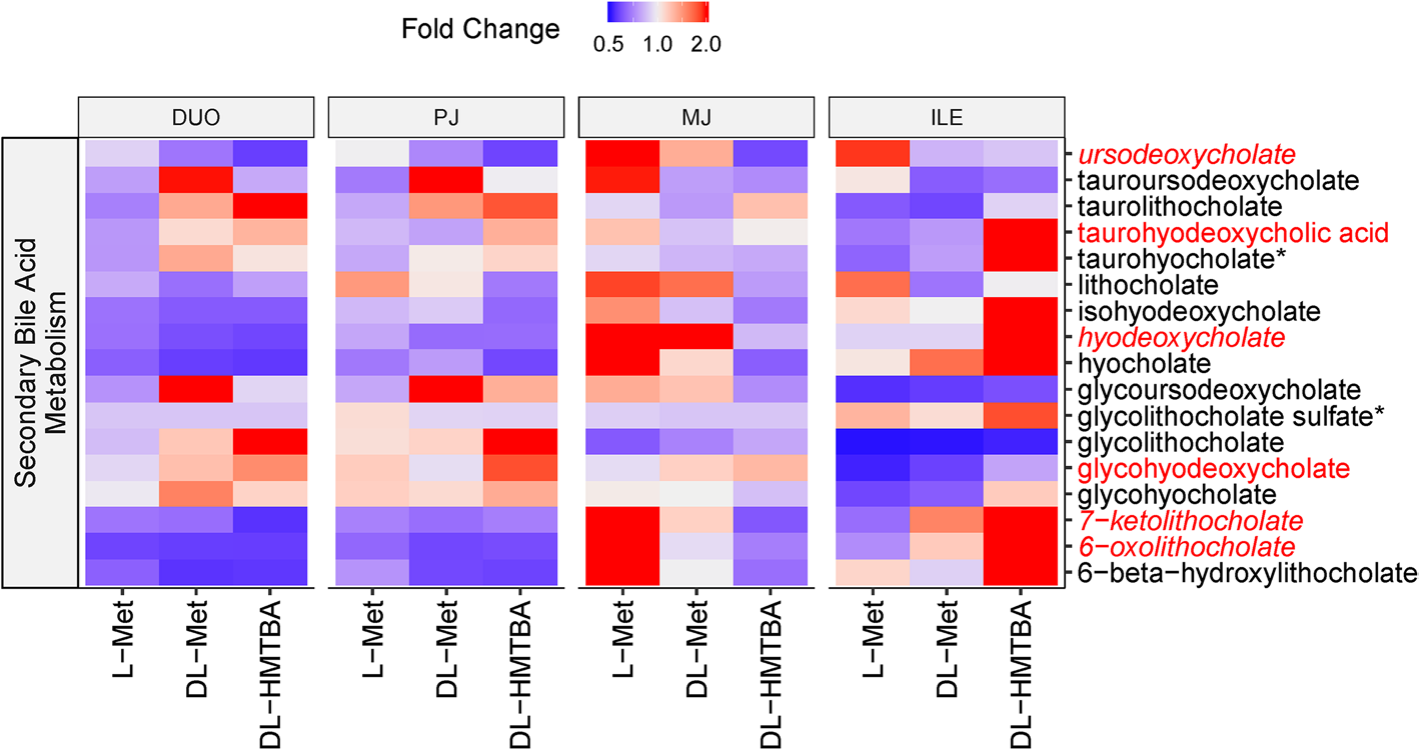
Heat map of the secondary bile acid metabolism pathway, showing the distribution of all measured pathway-related metabolites along duodenum (DUO), proximal jejunum (PJ), middle jejunum (MJ) and ileum (ILE) in pigs pre-fed with L-Met, DL-Met or DL-HMBTA. Blue color indicates lower abundance whereas red color indicates higher abundance of metabolites. Red writing of individual metabolites indicates a treatment effect or, if combined with italics, a region × treatment interaction (*P* < 0.10).

Several metabolites belonging to the secondary BA metabolism were significantly influenced by treatment or region × treatment interaction (*P* < 0.05; Fig. 4). These comprised 4 deoxycholate-derived metabolites plus 7-ketolitocholate and 6-oxolithocholate. The most obvious effect was a pronounced shift of secondary bile acids in DL-HMTBA-fed animals with lower values in MJ and higher values in ILE compared to the other two groups.

**Figure 5:**
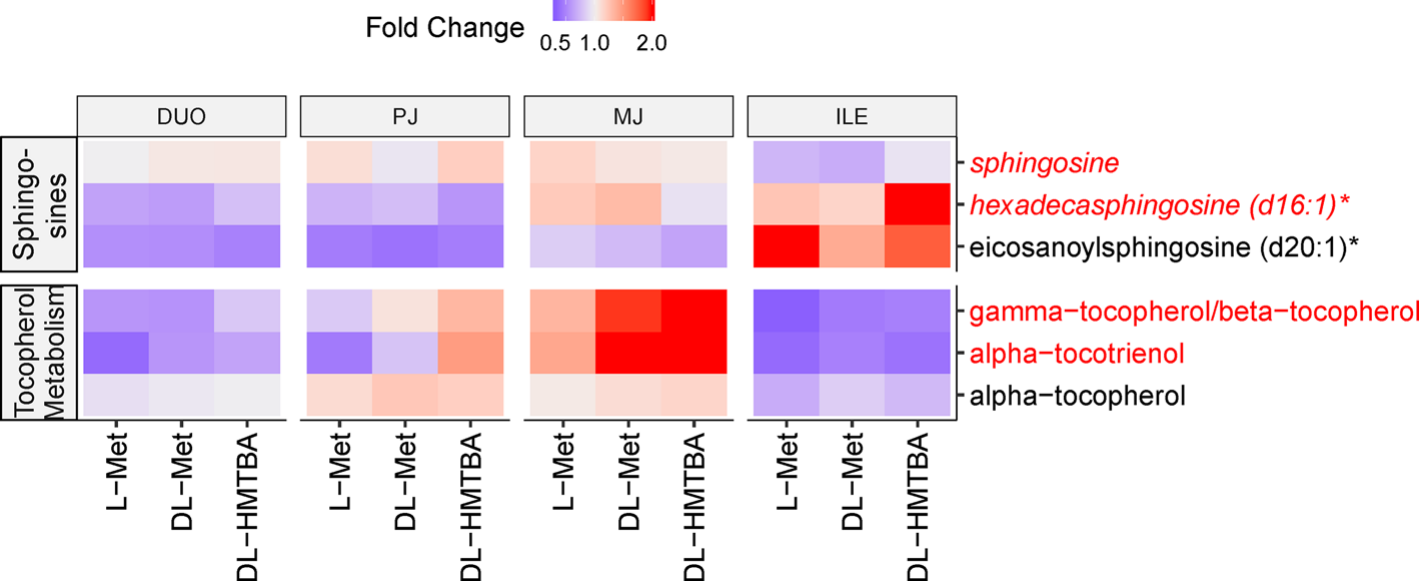
Heat map of sphingosines and the tocopherol metabolism pathway, showing the distribution of all measured pathway-related metabolites along duodenum (DUO), proximal jejunum (PJ), middle jejunum (MJ) and ileum (ILE) in pigs pre-fed with L-Met, DL-Met or DL-HMBTA. Blue color indicates lower abundance whereas red color indicates higher abundance of metabolites. Red writing of individual metabolites indicates a treatment effect or, if combined with italics, a region × treatment interaction (*P* < 0.10).

Alterations in the sphingosine pathway tended to be enriched by the dietary treatment (*P* = 0.064), with sphingosine and hexadecasphingosine (d16:1) being affected by a region × treatment interaction (*P* < 0.1; Fig. 5). The interaction was primarily based on higher abundance of these compounds in ILE of DL-HMTBA-fed animals.

A trend towards enrichment of treatment-dependent alterations in the tocopherol pathway (*P* = 0.064) was primarily based on higher abundance of γ-/β-tocoperol and α-tocotrienol in pigs supplemented with DL-Met and DL-HMTBA compared to L-Met (*P* < 0.1; Fig. 5).

**Figure 6:**
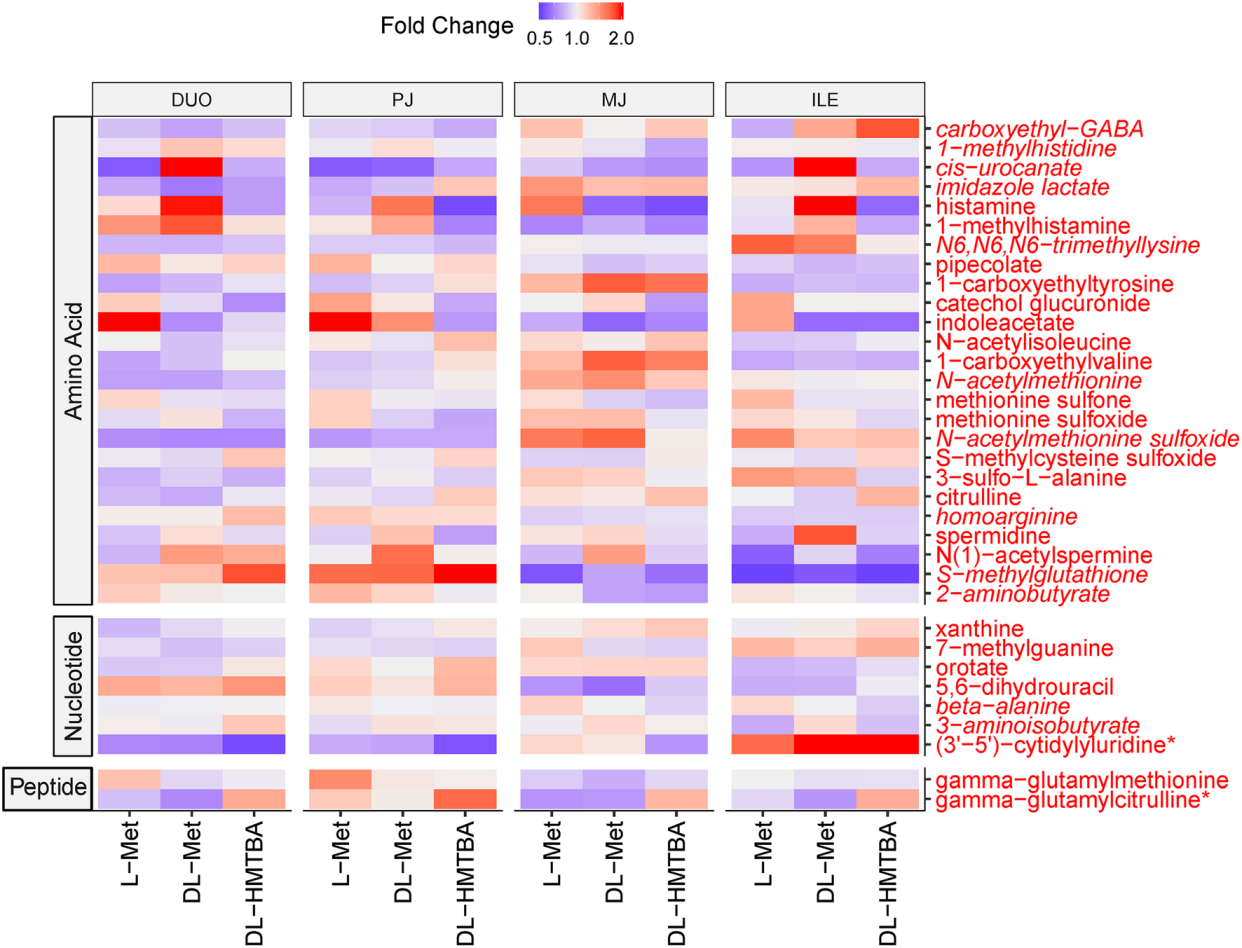
Heat map of individual metabolites from amino acid, nucleotide and peptide super-pathways that differed or tended to differ by treatment or region × treatment interaction (*P* < 0.10), the latter depicted by italics font. Depicted is the metabolite distribution along duodenum (DUO), proximal jejunum (PJ), middle jejunum (MJ) and ileum (ILE) in pigs pre-fed with L-Met, DL-Met or DL-HMBTA. Blue color indicates lower abundance whereas red color indicates higher abundance of metabolites.

**Figure 7:**
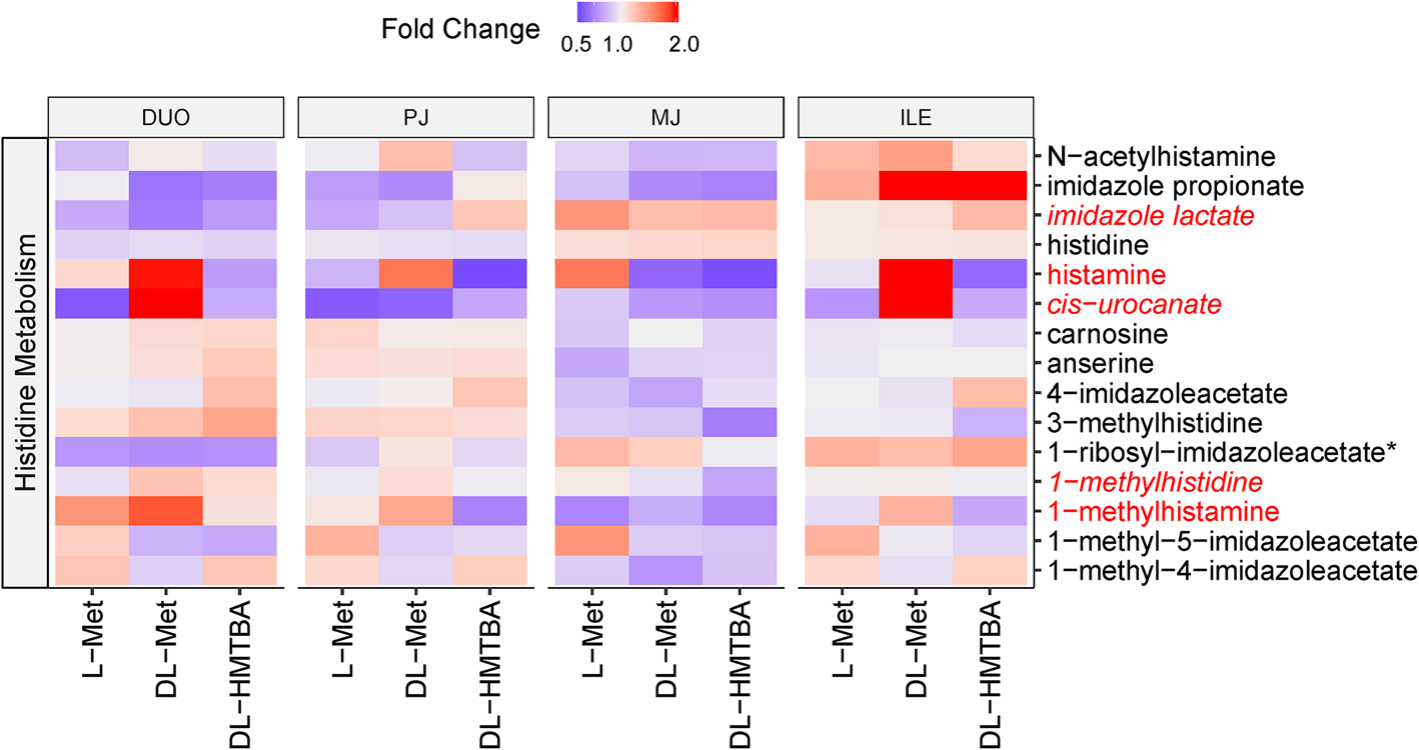
Heat map of the histidine metabolism pathway, showing the distribution of all measured pathway-related metabolites along duodenum (DUO), proximal jejunum (PJ), middle jejunum (MJ) and ileum (ILE) in pigs pre-fed with L-Met, DL-Met or DL-HMBTA. Blue color indicates lower abundance whereas red color indicates higher abundance of metabolites. Red writing of individual metabolites indicates a treatment effect or, if combined with italics, a region × treatment interaction (*P* < 0.10).

As dietary amino acid supplementation was the imposed treatment, the amino acid super pathway was looked at more closely. Out of 184 metabolites in the amino acid super pathway, 25 showed treatment effects, region × treatment interaction or trends thereof (*P* < 0.1; Fig. 6). However, the only amino acid pathway that tended to be enriched by changes due to the dietary treatment was histidine metabolism (*P* = 0.067; Table 1). The abundance of 1-methyhistidine and the histidine-degradation metabolites imidazole lactate and *cis*-urocanate was affected by region × treatment interaction, whereas histamine and 1-methylhistamine tended to be specifically enriched in DL-Met-supplemented animals across intestinal regions (*P* < 0.1; Fig. 7). Changes were not significantly enriched in the Met, cysteine, S-adenosylmethionine and taurine metabolism pathway (*P* = 0.13; Table 1); however, several oxidative stress-related metabolites of this pathway appeared selectively altered. Thus, pre-feeding a DL-HMTBA-containing diet induced a shift from Met-sulfoxide and Met sulfone (decreased abundance; *P* < 0.05) to S-methylcysteine sulfoxide (increased abundance; *P* < 0.1) across regions of the small intestine. The dipeptide γ-glutamylcitrulline showed higher contents in all intestinal regions of DL-HMTBA-supplemented pigs, whereas γ-glutamylmethionine was raised in L-Met-supplemented pigs in the proximal small intestine.

As Met is involved in nucleobase methylation^12,33^, several metabolites from the nucleotide metabolism super pathway were significantly changed in ANOVA analysis. However, no individual pathway of this super pathway was significantly enriched.

### Quantitative reverse-transcription polymerase chain reaction (qRT-PCR)

**Figure 8:**
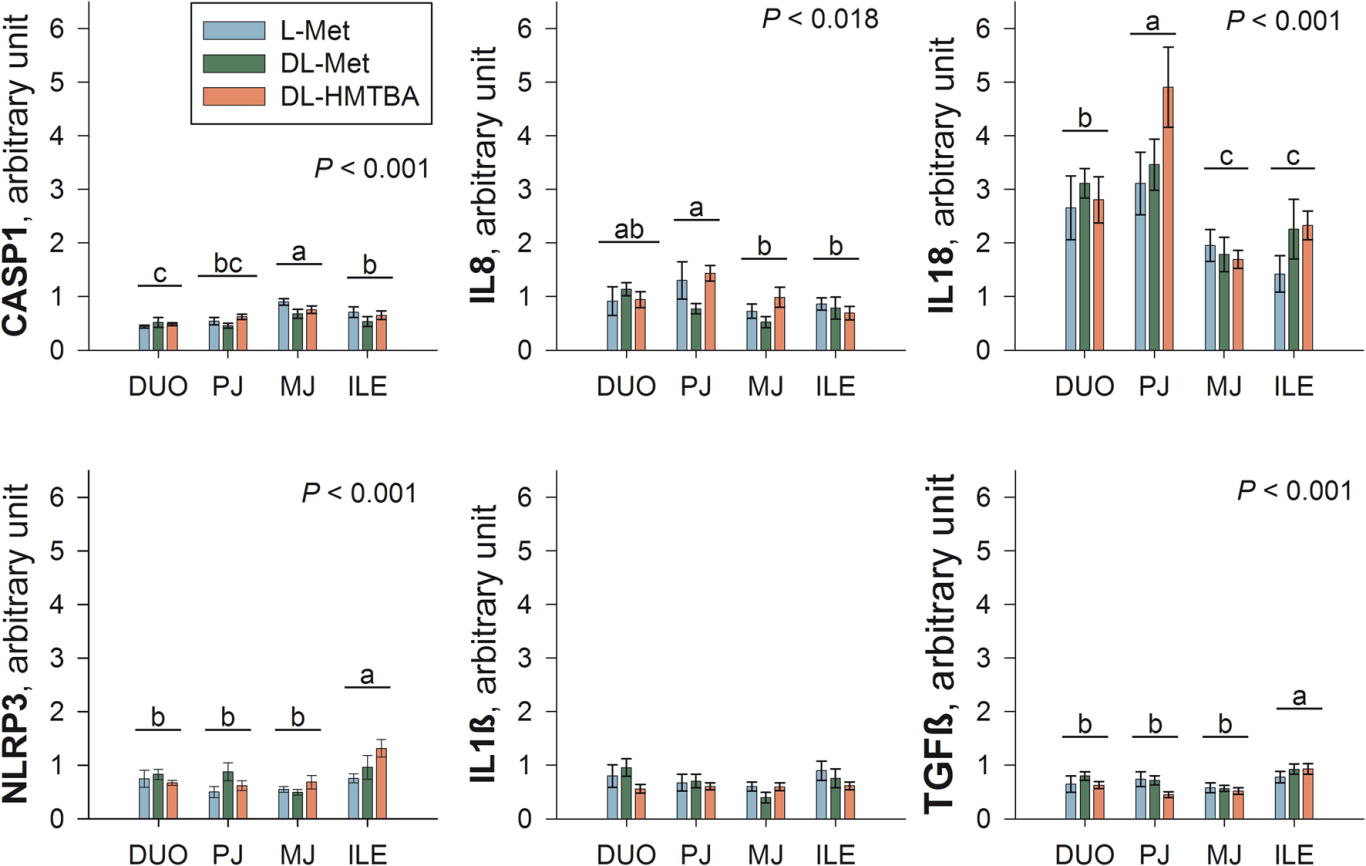
Calibrated normalized relative quantity expression of inflammation-related genes in duodenum (DUO), proximal jejunum (PJ), middle jejunum (MJ) and ileum (ILE) in DL-Met, L-Met and DL-HMTBA-fed pigs. Results are given as means with standard error. Significant differences between experimental groups are indicated by different letters a-c. *P*-Values of two-way ANOVA region main effects are given in each graph. There were no dietary treatment effects nor region × treatment interactions (*P* > 0.1).

Despite several regional differences, there were no main effects of diet and no interactions between tissue × diet on inflammation-related genes (*P* > 0.1; Fig. 8), namely *CASP1* (caspase 1), *NLRP3* (NLR family pyrin domain containing 3), *IL1β* (interleukin 1β), *IL8* (interleukin 8), *IL18* (interleukin 18) and *TGFβ* (transforming growth factor β).

## Discussion

**Figure 9:**
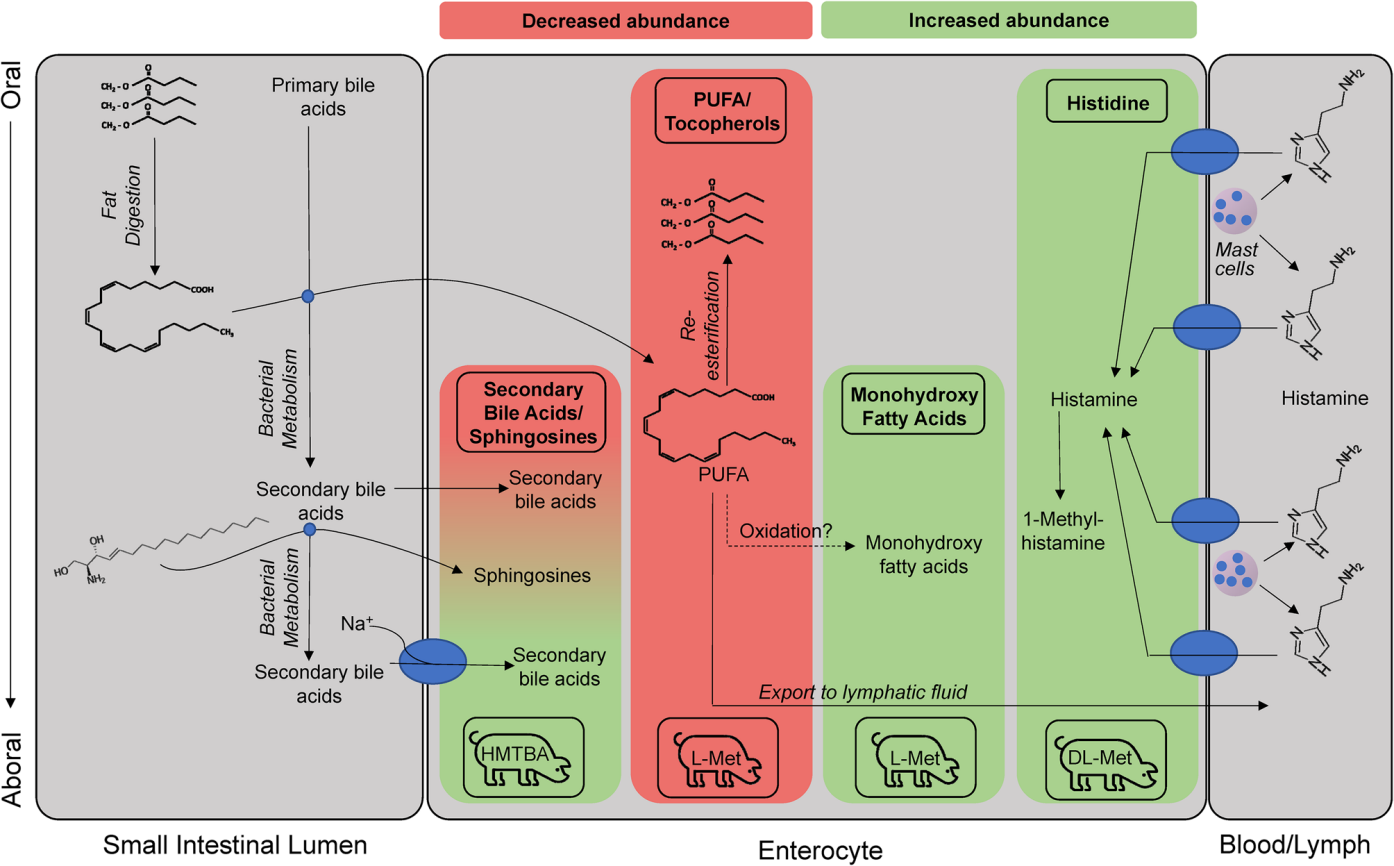
Illustration of key findings of the present study. The graphical sketch depicts the small intestinal lumen (in oral to aboral direction) as source of lipid metabolites and bile acids; as well as the interaction of lipid metabolites and bile acids during absorption (small blue circles). Metabolites within enterocytes are presented in a red or green panel area depending on whether their abundance was decreased or increased in the group of pigs cited at the bottom of the area. The right side of the sketch illustrates that the metabolite concentration within the enterocyte is also dependent on the capacity of exit into the blood or lymph stream or, as assumed for histamine, on its uptake across the basolateral membrane into enterocytes. Blue ellipses at the enterocyte membranes represent transport proteins. Further details are given and discussed in the text.

The present study is one of very few studies that investigated the effects of nutritional intervention on the complex metabolome of the intestinal epithelium. Numerous previous studies explored the effects of nutritional interventions on the metabolome in blood serum or plasma^34–37^, urine^38^, milk^39^, digesta^40^ or metabolic target organs like liver^41,42^, kidney^43^ and muscle^38^. However, only few studies have been published using porcine intestinal tissue^44^. The present study provides unique insights into dietary effects on the metabolome directly at the level of nutrient acquisition in the intestinal epithelium. The main findings are illustrated in Fig. 9.

In our previous investigation on samples of the same animals, we had identified increased absorptive capacity for L-Met after feeding the diet supplemented with DL-Met, especially, in the middle jejunum^23^. Thus, our initial expectation was that the different dietary sources of Met would also alter Met metabolites and possibly other related metabolites in the amino acid super pathway. This was based on the fact that L-Met is directly and intensely metabolized in the enterocyte^10,18,45^ whereas alternative Met sources like D-Met and DL-HMTBA require prior bioconversion into L-Met^21,22^. Surprisingly however, different dietary Met sources targeted primarily at the lipidome, with only small and selective effects on single metabolites in the amino acid super pathway. Apart from trends for histidine metabolism, these effects did not add to an enrichment of a specific amino acid metabolism pathway.

### Methionine, cysteine, S-adenosylmethionine and taurine metabolism

Met is well known for its antioxidative capacity^46,47^. It is the precursor of molecules implemented in antioxidative defense like cysteine and its metabolites glutathione and taurine^17,46^. Met itself can also be oxidized and reduced back to Met by Met sulfoxide reductase^46,47^. In the present study, direct oxidation products of Met (Met sulfoxide and Met sulfone) were lower in pigs fed diets supplemented with DL-HMTBA compared to pigs supplemented with DL-Met and, especially, L-Met. However, the lower levels of Met sulfoxide and Met sulfone cannot be translated linearly into lower oxidative stress in the small intestine of DL-HMTBA-fed pigs. Albeit there could be support of such postulate from few studies that suggest particular benefits of DL-HMTBA for the antioxidative capacity of the body^30,31^, there is disagreement in the literature. More recent research indicates that there are no consistent differences in antioxidant potential between DL-HMTBA and DL-Met when sufficient Met + cysteine is provided in the diet^48–50^. Other studies have even observed beneficial effects of either DL-Met or L-Met supplementation on antioxidant status compared with DL-HMTBA^51–53^. Thus, the higher levels of Met sulfur oxidation products (which may include both D-Met and L-Met sulfur oxidation products) in pigs supplemented with DL-Met and L-Met could be seen as a consequence of higher Met turnover in the intestinal epithelium during absorption. Vice versa, a decreased turnover of Met in the small intestine of pigs fed DL-HMTBA may explain the observed trend for higher abundance of alternative sulfur oxidation products (S-methylcysteine sulfoxide) in their small intestinal segments. As such, the shift from Met sulfur oxidation products to S-methylcysteine oxidation products in pigs fed DL-HMTBA bears similarities to the shift from the dipeptide γ-glutamylmethionine to γ-glutamylcitrulline; both phenomena appear due primarily to a lower turnover of Met in the intestinal epithelium of DL-HMTBA-fed pigs.

### Lipid metabolism

Having acknowledged that the observed differences in Met sulfur oxidation products have limited value for the assessment of the oxidative status in enterocytes of pigs in the three feeding groups, changes in the intestinal epithelial lipidome could potentially be interpreted as diet-induced changes in epithelial oxidative status. However, such changes were not evident between L-Met and DL-Met vs. DL-HMTBA but between L-Met vs. DL-Met and DL-HMTBA. This postulate is based on coordinated changes in three metabolic pathways. Pigs receiving a diet supplemented with L-Met had (1) lower concentrations of PUFA in epithelial cells of all small intestinal segments, (2) higher levels of monohydroxy fatty acids in the distal small intestinal segments (MJ and ILE) and (3) lower levels of certain tocopherol metabolites across all small intestinal epithelia. As PUFA are highly susceptible to oxidation, their reduced levels in pigs fed the L-Met diet might indicate higher oxidative stress^54^. The oxidation products of PUFA would be monohydroxy fatty acids, which were increased in the distal small intestinal segments of pigs supplemented with L-Met. However, the altered metabolites in the monohydroxy fatty acid pathway, mainly included 2-hydroxyl products of saturated fatty acids which are intentionally produced by the body for synthesis of mammalian sphingolipids^55,56^. It was shown that 2-hydroxy fatty acids are highly abundant in intestine of developing rats, constituting the major fatty acid fraction in intestinal glucosylceramides^57^. Ceramides, a subclass of sphingolipids, are known for their inflammatory response signaling^58^ and induction of apoptosis^58,59^. It was also shown that ceramides containing monohydroxy fatty acids induce stronger pro-apoptotic signaling than non-hydroxy ceramides^60^. Importantly, however, the ceramide pathway was not significantly altered in our study (Pathway Heat Map in Supplementary Table 1, columns AF through AQ), which does not generate a link to inflammation. On the contrary, 2-hydroxy fatty acids may have a beneficial stabilizing effect on the intestinal epithelial cell membrane, which makes enterocytes more resistant to environmental stress^55,61^. Additionally, the glutathione metabolism pathway was not affected by treatment. Therefore, the observed accumulation of 2-hydroxy fatty acids and the decreased abundance of PUFA in L-Met-supplemented pigs is most probably neither linked to generalized oxidative stress nor to an inflammatory reaction.

This is further supported by the changes in the tocopherol metabolism pathway. Although reduced levels of tocopherols could indicate higher oxidative stress, changes were restricted to low-abundance tocopherol metabolites whereas α-tocopherol, which is known as the dominating tocopherol metabolite in enterocytes^7^, was not altered.

As an alternative explanation for the lower levels of epithelial PUFA in L-Met-supplemented pigs, it should also be considered that those lower levels might simply result from altered lipid digestion and absorption. Such postulate has partial support from parallel decreases in several long-chain fatty acids, monoacylglycerols and lysophospholipids in small intestinal epithelia of pigs supplemented with L-Met compared to DL-Met that were indicated by individual group comparisons but did not penetrate to overall significance on ANOVA or pathway enrichment analysis (Pathway Heat Map in Supplementary Table 1, columns AF through AQ). The intraepithelial concentration of a given fatty acid is the result of luminal lipid digestion followed by cellular lipid uptake, re-esterification and export of lipids to the lymphatic fluid^62^. Each of these steps can have an impact on its intraepithelial concentration.

### Bile acid metabolism

Bile acids could influence PUFA digestion and absorption as Rohrl et al.^63^ showed that cellular uptake of PUFA was selectively improved by micellization of an oil. Changes in BA metabolism were, indeed observed after applying the three dietary treatments in the present study. However, these changes were not compatible with a decreased PUFA availability in L-Met-supplemented pigs. We observed even higher contents of secondary bile acids in MJ in L-Met-supplemented pigs compared to the DL-Met and, especially, the DL-HMTBA group. The major change in BA metabolism, however, occurred in the DL-HMTBA group, where we observed very high levels of secondary bile acids in ILE. Secondary BA derive from bacterial metabolism of primary BA. Following absorption, secondary BA are treated like primary BA, i.e., they are subject to taurine or glycine conjugation in the liver to decrease their toxicity and increase their water solubility before secretion into duodenum^64^. Conjugated primary and secondary BA are actively (re-)absorbed by the apical sodium dependent bile acid transporter (ASBT), predominantly, in ILE^65^. Besides active transport, deconjugated primary or secondary BA may pass the intestinal barrier passively in the small intestine and colon^65^. The latter challenges epithelial cell homeostasis; thus, deconjugated secondary BA have been associated with increased risk for colon cancer, because deconjugation of BA and synthesis of secondary BA occurs primarily in the large intestine^66,67^. Nonetheless, anaerobic and facultative anaerobic bacteria may perform deconjugation and hydroxyl group oxidation of conjugated BA already in the small intestine^68,69^. Thus, dysbiosis can have a major impact on secondary BA metabolism. It was shown in people and dogs that individuals with active inflammatory intestinal diseases display an increased fecal content of primary BA because of decreased small intestinal absorption and decreased microbial conversion of BA^70,71^. It is thus perceivable that the antibacterial effects of DL-HMTBA demonstrated in a porcine intestinal model in vitro^72^ and in chicken’s cecum in vivo^73^ may delay small intestinal production of secondary BA. This could explain the observed shift of secondary, especially deconjugated, BA in DL-HMTBA-supplemented pigs with lower levels in MJ and higher levels in ILE. One alternative explanation might be that HMTBA alters the active uptake of conjugated and/or the passive uptake of unconjugated BA in the distal small intestine.

The observed changes in bile acid metabolism appear, in principle, compatible with a delayed metabolism of sphingomyelins to sphingosines, where a similar shift in sphingosine metabolites from MJ (lower levels) to ILE (higher levels) was observed in the DL-HMTBA-fed group. It has been shown previously that the release of sphingosine from sphingomyelins by intestinal alkaline sphingomyelinase is strictly dependent on the presence and species of BA^74,75^.

### Histidine metabolism

As mentioned earlier, histidine metabolism was the only amino acid metabolism pathway where diet-induced alterations were enriched in the present study. Indeed, Met is involved in histidine metabolism, as S-adenosylmethionine is a methyl group donor for the production of 1- or 3-methylhistidine and 1-methylhistamine^13^. Histidine can be decarboxylated to histamine, which is well known for its involvement in inflammation and a variety of diseases^76–78^. The enrichment pattern of the histidine metabolism pathway was not very clear and included region × treatment interactions for two histidine degradation products (imidazole lactate and *cis*-urocanate), indicating that dietary Met sources directly influence histidine related metabolites in selected intestinal segments. One very consistent finding was that histamine and its degradation product, 1-methylhistamine, tended to be specifically enriched in DL-Met-supplemented animals. The intestine is rich in mast cells as a primary source of histamine^77,79^, possibly implying that epithelial mast cells or their histamine production was stimulated by a DL-Met-containing diet. Alternatively, it has been shown in pigs that the colonocytes take part in clearing histamine from blood, most likely via organic cation transporter 1, followed by biotransformation by histamine N-methyltransferase (to 1-methylhistamine) and diamine oxidase^80^. Although those previous results were obtained in colon, basolateral organic cation transporters^81^ and both catabolic enzymes are also present in the small intestine^82,83^. Their stimulation by a DL-Met-containing diet could thus explain higher intraepithelial concentrations of histamine and 1-methylhistamine. In support of such postulate, supplementation of DL-Met was shown to induce increased absorption of L-Met in the same pigs as used in the present study^23^.

### Inflammatory status

The alterations in histamine metabolism and the earlier discussed alterations in PUFA and secondary BA metabolism could potentially imply diet-induced changes in inflammatory status. Histamine is a key facilitator of the inflammatory response^76,77^ and PUFA are also closely related to inflammation as they are precursors of lipid mediators like eicosanoids^84^. To elucidate a possible relevance of these metabolite changes for inflammatory status, we complemented the metabolomics analysis with qRT-PCR experiments to determine the expression of genes that are related to different inflammatory pathways. The main outcome of the qPCR experiments was that mRNA expression of all investigated genes but *IL1β* differed among intestinal segments. However, no gene expression differences were observed between feeding groups, suggesting either similar or no effects of the three dietary supplements on intestinal inflammatory status.

## Conclusion

This unique study on the effects of three relevant Met supplements on intraepithelial metabolite concentrations revealed only minor diet-dependent changes of the overall metabolome in epithelia of four small intestinal segments. However, in-depth analysis showed coordinated changes in the PUFA, monohydroxy fatty acid and tocopherol metabolism pathways in pigs supplemented with L-Met. This may suggest either increased oxidation of PUFA and/or decreased absorption of PUFA. The latter requires further investigations as none of these options was clearly supported by changes in other oxidative stress- and lipid digestion-related metabolites. Furthermore, pigs supplemented with DL-HMTBA showed a pronounced shift of secondary BA and sphingosine metabolites from MJ to ILE, possibly indicating altered microbial BA metabolism and/or epithelial transport. Importantly, the observed changes of some potentially inflammation-related metabolites (PUFA and BA), as well as the observed changes in histidine/histamine metabolism in DL-Met-supplemented pigs, had no measurable impact on the inflammatory status of the intestinal epithelia as evidenced by similar expression of several inflammation-related genes.

## Materials and methods

### Ethics declaration

All experiments involving pig handling and treatments were approved by the local authorities responsible for animal care and use approval, the ‘Landesamt für Gesundheit und Soziales Berlin’ (LaGeSo Reg. No. T 0264/15), which included assessment by the advising ethics committee (Tierversuchskommission Berlin). All methods were performed in accordance with German and European guidelines and regulations regarding animal protection and welfare. The study is reported according to the ARRIVE guidelines.

### Animals and diets

Details on the animal trial, sample size and experimental diets have been published previously^23^ and are available with open access under https://doi.org/10.1093/jn/nxaa115. In brief, 27 Danbred × Piétrain male castrated pigs were purchased from a commercial farm. Pigs were kept in stainless steel frames with concrete floor (1.9 m × 1.9 m) at the Institute of Animal Nutrition, Freie Universität Berlin, Germany. They were ~ 10 wks old and weighed ~ 25 kg at the start of the trial. The trial was performed in three consecutive runs with 9 pigs per run randomly allocated into 3 groups. Groups received a diet (10.3 MJ NE/kg, 18% crude protein) based on corn, soybean meal, barley, and peas, which met requirements of the National Research Council (NRC)^85^ and contained sufficient concentrations of all amino acids except standardized ileal digestible Met + cystine (0.46%). To provide adequate standardized ileal digestible Met + cystine (0.67%), diets were supplemented with one of the following Met supplements: 0.21% DL-Met, 0.21% L-Met or 0.31% DL-HMTBA. DL-HMTBA was provided at a higher dietary concentration to account for its lower bioefficacy (~ 70% of Met)^1^. During the entire pre-feeding period, piglets had ad libitum access to water and feed.

Animals were killed after being on the diet for at least 10 d. Euthanasia was performed at 4 h after the morning feeding that was provided at 6.00 a.m. Pigs were sedated using 20 mg/kg bodyweight of ketamine hydrochloride (Ursotamin; Serumwerk Bernburg AG, Bernburg, Germany) and 2 mg/kg bodyweight of azaperone (Stresnil; Jansen-Cilag, Neuss, Germany). Pigs were then killed by intracardial injection of 10 mg/kg bodyweight of tetracaine hydrochloride, mebezonium iodide and embutramide (T 61; Intervet, Unterschleißheim, Germany). Euthanasia by drug administration was preferred over conventional slaughter by captive bolt stunning or electroshock and exsanguination as the latter initiates profound sympathetic activation with release of adrenaline, noradrenaline and cortisol together with histamine^86^.

### Tissue sampling

After killing, a mid-line incision was made, and samples were obtained from DUO, PJ, MJ and ILE. The *tunica muscularis externa* was mechanically removed. Samples of the *tunica mucosa* were snap-frozen in liquid nitrogen and subsequently stored at − 80 °C for metabolomics analyses. *Tunica mucosa* samples for qRT-PCR were preserved in RNA*later* (Sigma Aldrich, St. Louis, Missouri, USA) and stored at 4 °C overnight, followed by storage at—20 °C until analyses.

### Metabolomics

A complete set of sample aliquots (100 mg to 300 mg of each sample) were shipped to Metabolon Inc. (Morrisville, NC, United States) on dry ice, where samples were kept at − 80 °C until analysis by ultrahigh performance liquid chromatography and tandem mass spectroscopy (UPLC-MS/MS). As samples were analyzed in Metabolon’s semi-automated work flow without any prior communication on possible outcomes, sample blinding was not required. The proprietary technology of Metabolon Inc. includes addition of several recovery standards during sample preparation for quality control purposes. After protein precipitation with methanol, extracts were divided into four fractions, of which two were used for two separate reverse phase (RP) UPLC-MS/MS methods with positive ion mode electrospray ionization (optimized for either hydrophobic or hydrophilic compounds), one for an RP-UPLC-MS/MS method with negative ion mode electrospray ionization and one for hydrophilic interaction liquid chromatography (HILIC)/UPLC-MS/MS with negative ion mode electrospray ionization. Analyses were run on Waters ACQUITY UPLC (Waters, Eschborn, Germany) and Thermo Scientific Q-Exactive (Waltham, MA, United States) high resolution/accurate mass spectrometer. MS was combined with heated electrospray ionization (HESI-II) and an Orbitrap mass analyzer operated at 35,000 mass resolution.

Following chromatographic mass spectral analyses, raw data was extracted, peak-identified and quality control-processed using Metabolon’s hardware and software. Identification of compounds was achieved by comparison to Metabolon’s compound library that includes retention time/index, mass-to-charge ratio and chromatographic data (including MS/MS spectral data). Biochemical identifications were based on (1) retention index with a narrow retention time/index (RI) window of the proposed identification, (2) accurate mass match to the library ± 10 ppm and (3) MS/MS forward and reverse scores between the experimental data and authentic standards. The MS/MS scores were based on a comparison of the ions present in the experimental spectrum to the ions present in the library spectrum containing ~ 5,300 commercially available purified standard compounds. Peaks were quantified using area-under-the-curve. A data normalization step was performed for studies spanning several analytical days. Each compound was corrected in run-day blocks by registering the medians to equal one and normalizing each data point proportionately.

### Statistical analysis of metabolomics data

Statistical analysis was run by Metabolon Inc. as part of the provided service. Analyses included log transformation of data and imputation of missing values prior to repeated measures ANOVA with the fixed factors region (DUO, PJ, MJ and ILE), treatment (L-Met, DL-Met and DL-HMTBA) and the two-way region × treatment interaction. Collection cohort was incorporated as a random effect.

Metabolites that showed significant treatment effects or trends towards a treatment effect and metabolites that showed region × treatment interactions were incorporated into a pathway enrichment analysis based on Metabolon’s pathway map using the chi square test implementation of base R. No multiple-testing correction was performed to increase sensitivity over specificity for the pathway enrichment analysis. Results were considered significant at *P* ≤ 0.05, with tendencies towards significance defined at 0.05 < *P* ≤ 0.10.

Principal component analysis using the prcomp function from base R was performed to identify and visualize similarities in data set structures among data of different regions and treatments.

### RNA extraction and control of RNA integrity and purity

The Nucleospin RNA kit (Macherey & Nagel, Dueren, Germany) was used for RNA extraction according to the manufacturer’s instruction. RNA quality was checked using lab-on-a-chip electrophoresis by Agilent 2100 Bioanalyzer (Agilent Technologies Inc., Santa Clara, CA, United States). Four samples were excluded because of poor RNA quality (RIN < 6). All other samples had satisfactory RNA quality (RIN ≥ 6). Purity of RNA was verified by NanoPhotometer P330, Version 1.0 IMPLEN (Implen GmbH, Munich, Germany) with absorption ratios ≥ 2.0 at 260/280 nm and 260/230 nm. NanoPhotometer P330 was also used to determine RNA concentration.

### cDNA synthesis

cDNA was synthesized from a 1,000 ng/μl RNA template using iScript cDNA Synthesis Kit and iCycler iQ (both from Bio Rad, Hercules, CA). A -RT (reverse transcriptase) sample was prepared without enzyme mix to be used as negative control in qRT-PCR. The thermal cycler protocol was run according to iScript cDNA Synthesis Kit instructions (priming at 25 °C for 5 min, reverse transcription at 46 °C for 20 min, reverse transcriptase inactivation at 95 °C for 1 min). After reverse transcription, cDNA was diluted 1:10 with ddH_2_O (final concentration 5 ng/μl).

### Quantitative reverse-transcription polymerase chain reaction

Six inflammation-related genes were chosen for qRT-PCR analysis, including *CASP1*, *NLRP3*, *IL1β*, *IL8*, *IL18* and *TGFβ*. The following unregulated housekeeping genes were used for normalization: *GAPDH* (glycerinaldehyde-3-phosphate dehydrogenase) and *ACTB* (β-actin). The unregulated expression of the used housekeeping genes was verified statistically (*P* > 0.05; Supplementary Fig. 1). Primers were purchased from Eurofins Genomics (Ebersberg, Germany). Primer sequences are listed in Table 2.

**Table 2:** Accession numbers and primer sequences of genes amplified by qRT-PCR. Primer sequences are taken from Loss et al.^87^.

Gene	Accession no	Primer	Sequence 5’–3’ (nt)
*NLRP3*	NM_001256770.2	Fwd	AGCACATTCCAGTGCATCAAAG
		Rev	CCTGGTGAAGCGTTTGTTGAG
*IL-1β*	NM_214055.1	Fwd	CCTCCTCCCAGGCCTTCTGT
		Rev	GGGCCAGCCAGCACTAGAGA
*IL-8*	X61151.1	Fwd	GGCAGTTTTCCTGCTTTC
		Rev	CAGTGGGGTCCACTCTC
*IL-18*	AF191088.1	Fwd	ACGATGAAGACCTGGAATCG
		Rev	GCCAGACCTCTAGTGAGGCTA
*TGFβ*	NM_214015.2	Fwd	TGACCCGCAGAGAGGCTATA
		Rev	CATGAGGAGCAGGAAGGGC
*CASP1*	NM_214162	Fwd	CTCTCCACAGGTTCAXAATC
		Rev	GAAGACGCAGGCTTAACTGG
*GAPDH*	DQ178124	Fwd	ACTCACTCTTCTACCTTTGATGCT
		Rev	TGTTGCTGTAGCCAAATT
*ACTB*	DQ178122	Fwd	TCTGGCACCACACCTTCT
		Rev	TGATCTGGGTCATCTTCTCAC

qRT-PCR was performed using iQ SYBR Green Supermix Kit (BioRad, Hercules, CA, United States) in assay volumes of 15 μL with 5 μL cDNA. Reactions were carried out in 384-well plates (BioRad, Hercules, CA, United States) with three replicates per reaction. For negative control, ddH_2_O and –RT samples were used. A pool sample, originating from 12 samples from the different intestinal regions of pigs from the three different groups, was included on each plate and later used as inter-run calibrator for calibration. For cDNA amplification, ViiA7 (Applied Biosystems/Life Technologies, Waltham, MA, United States) was used. Run method included 40 amplification cycles (95 °C for 12 s, 60 °C for 1 min). Thresholds were manually set to 1,500.

### Statistical analysis of qRT-PCR data

Cycle threshold (C_t_) values were normalized to the mean value of the two housekeeping genes and calibrated to the pool sample using the ΔΔC_t_ method^88^. Calibrated normalized relative quantity expression data was used for statistical analysis. 19 Outliers were identified using a Grubbs test^89^ at a significance level of 0.05 and removed from the dataset. Five values were excluded due to operational errors (*n* = 7–9). Sigma Plot 11.0 (Systat Software GmbH, Erkrath, Germany) was used for statistical analyses. A two-way ANOVA with the fixed factors intestinal tissue region (DUO, PJ, MJ and ILE), dietary treatment (L-Met, DL-Met and DL-HMTBA) and the two-way region × treatment interaction was performed, followed by Student-Newman-Keul’s posthoc test. Results were considered significant at *P* ≤ 0.05.

## Supplementary Information

Supplementary Figure 1.

Supplementary Table 1.

## Data Availability

All data generated or analyzed during this study are included in this published article and its supplementary information files.
