# Supplementary material for: Dietary methionine source alters the lipidome in the small intestinal epithelium of pigs
Source: Sci Rep. 2022 Mar 22;12:4863. doi: 10.1038/s41598-022-08933-3 (PMC8941097; doi:10.1038/s41598-022-08933-3)
Supplement: Supplementary file 1 — Supplementary Figure 1. [file 41598_2022_8933_MOESM1_ESM.pdf]

Schermuly et al. (2022):

Dietary methionine source alters the lipidome in the small intestinal epithelium of pigs.

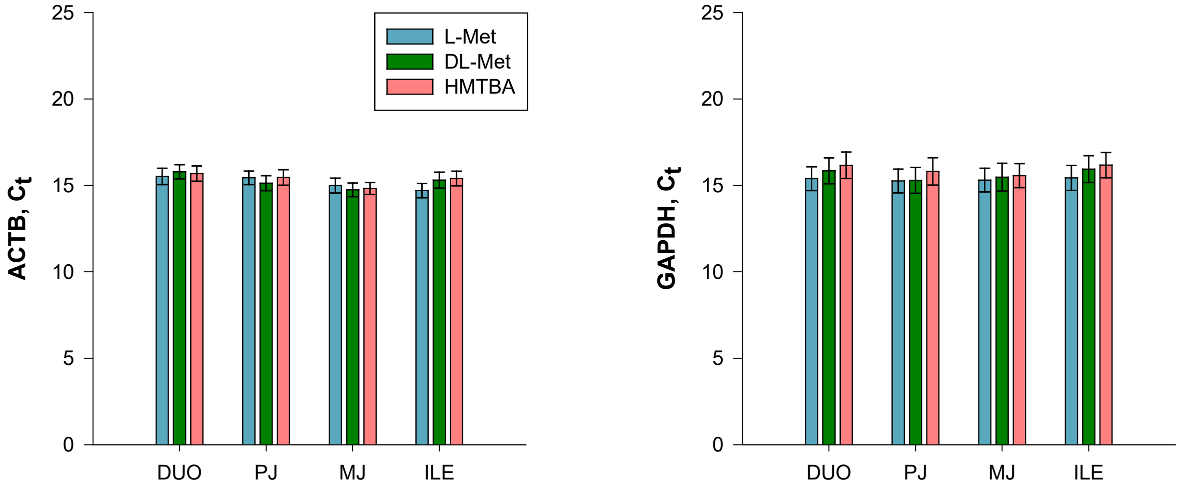

Supplementary Fig. 1. Validation of unregulated expression of housekeeping genes used for normalization of qRT-PCR data. Pigs were fed diets supplemented with different methionine (Met) sources, i.e., 0.21% L-Met, 0.21% DL-Met and 0.31% DL-2-hydroxy-4-(methylthio)butanoic acid (DL-HMTBA). ACTB, β-actin; GAPDH, glycerinaldehyde-3-phosphate dehydrogenase
